# Subretinal Therapy: Technological Solutions to Surgical and Immunological Challenges

**DOI:** 10.3389/fmed.2022.846782

**Published:** 2022-03-23

**Authors:** Reza Ladha, Laure E. Caspers, François Willermain, Marc D. de Smet

**Affiliations:** ^1^Departments of Ophthalmology, Centre Hospitalier Universitaire Saint-Pierre and Brugmann, Brussels, Belgium; ^2^Université Libre de Bruxelles, Brussels, Belgium; ^3^Department of Ophthalmology, Leiden University, Leiden, Netherlands; ^4^Preceyes B.V., Eindhoven, Netherlands; ^5^MIOS SA, Lausanne, Switzerland

**Keywords:** subretinal delivery, gene therapy, cell therapy, immune response, retina, ocular robotics

## Abstract

Recent advances in ocular gene and cellular therapy rely on precisely controlled subretinal delivery. Due to its inherent limitations, manual delivery can lead to iatrogenic damage to the retina, the retinal pigment epithelium, favor reflux into the vitreous cavity. In addition, it suffers from lack of standardization, variability in delivery and the need to maintain proficiency. With or without surgical damage, an eye challenged with an exogenous viral vector or transplanted cells will illicit an immune response. Understanding how such a response manifests itself and to what extent immune privilege protects the eye from a reaction can help in anticipating short- and long-term consequences. Avoidance of spillover from areas of immune privilege to areas which either lack or have less protection should be part of any mitigation strategy. In that regard, robotic technology can provide reproducible, standardized delivery which is not dependent on speed of injection. The advantages of microprecision medical robotic technology for precise targeted deliveries are discussed.

## Introduction

There are a growing number of hereditary and degenerative diseases of the retina that are the target of pre-clinical or clinical research using both genetic vectors or cell based therapies (1, 2).

The use of viral vectors in humans has centered on understanding the mechanisms of disease leading to a progressive loss of photoreceptors through apoptotic and non-apoptotic mechanisms, as well as the means to correct the defect (3, 4).

These treatments are dose and volume dependent, somewhat similar to pharmacologic therapy. Hence, optimizing delivery, devising a reproducible, standardized method to appropriately target retinal tissues and layers can improve on the experimental and clinical results by reducing the loss of vectors from the intended target and a reduce risk of an immune response.

It has been assumed that current available surgical techniques are sufficient and reliable in the hands of surgeons, many of whom, have limited training in performing this type of surgery (5, 6). Several lines of evidence indicate that this may not be the case (7–9).

**Figure 1:**
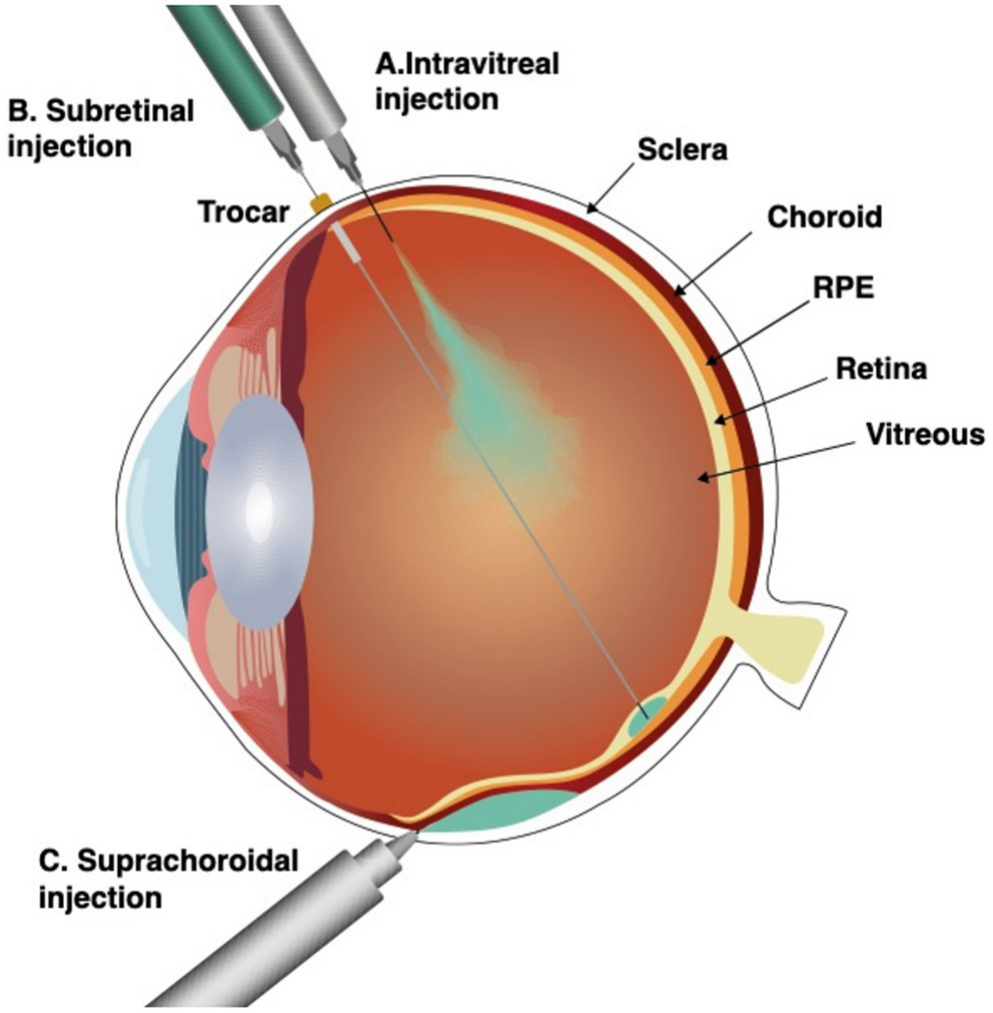
Surgical approaches for subretinal delivery. Intravitreal injection (A), subretinal injection (B), and suprachoroidal injection through a microneedle (C). Intravitreal injection fills the vitreous body and exposes all intraocular surfaces in contact with the vitreous to the vectors or cells. Subretinal injection, delivers the therapeutic suspension immediately under the sensory retina, into the subretinal space, a virtual space between the photoreceptors and the retinal pigment epithelium (RPE). Suprachoroidal injection by a microneedle, delivers the therapeutic suspension into to the suprachoroidal space, a virtual space between choroid and sclera.

Three surgical approaches are currently proposed to reach the retina: direct intravitreal injection which fills the core of the eye and exposes all intraocular surfaces in contact with the vitreous to the vectors or cells, or by injecting the suspension under the sensory retina either by a transvitreal route or reaching the subretinal space *via* the suprachoroidal space, a virtual space between the sclera and deeper eye structures (Figure 1) (10, 11). Each approach brings a unique set of advantages and drawbacks which should be considered in the context of the chosen therapeutic strategy and adapted to each specific disease being targeted (Table 1).

**Table 1:** Advantages and drawbacks of surgical approaches for retinal delivery.

**Intraocular location and approach**	**Target specific retinal layer**	**Area of transfection or effect**	**Risk of reflux and location**	**Risk of surgical complication**	**Risk of immune response**
Subretinal *via* PPV	Yes	Limited beyond area of retinal detachment	Yes into vitreous	Yes, area of retinal manipulation and related to surgical technique	Limited unless breech of barrier or reflux.
Subretinal *via* choroid	Yes	Limited effect beyond area of retinal detachment	Yes into the choroid	Intravitreal injection, choroidal hemorrhage	Systemic immune response or granuloma formation possible
Intravitreal	No but better reach of ganglion cells and other superficial cells of the inner retinal/ciliary body surface	All exposed tissues to the vitreous and some exposure of anterior chamber structures	At sclerotomy site	Limited—vitreous hemorrhage	Immune response likely mostly mild
Suprachoroidal	No	Limited if viscous fluid Disseminated to large are of suprachoroidal space with non-viscous fluid	External (subconjunctival). Cannot judge amount delivered	Yes, Injection into the vitreous cavity or choroidal hemorrhage	Yes—risk of systemic immune response

Accessing the eye by intravitreal injections or *via* the suprachoroidal space was proposed to circumvent the difficulties inherent with a transvitreal approach for subretinal delivery.

This review will address the challenges, advantages, and limitations of the current methods used to target the subretinal space. We will explore the role of robotic technology in understanding, optimizing and delivering gene and cell products to the subretinal space and propose solutions that make use of this novel technology.

## Challenge of Controlled Subretinal Delivery

### Human Limitations

In contrast to subretinal maneuvers in common usage such as tPA injections for submacular hemorrhages or the removal of subretinal fibroproliferative membranes, the specific challenge in subretinal gene therapy is that delivery should be confined to this space while avoiding a breach of the external blood ocular barrier or reflux into the vitreous cavity. Delivering precisely to the subretinal space without breaching Bruch's membrane and entering the choroid is challenging. The retina lacks elasticity which implies that any lateral movement of the needle tip or any attempted re-insertion will be associated with a high risk of widening the retinotomy, leading to reflux. Hence a reflux free delivery of a defined product to the subretinal space is rarely achieved using current techniques, even in healthy retina. It is even less likely in thinned, atrophic or scarred retina.

Physiologic hand tremor challenges surgeons who must deliver gene/cell solutions over a substantial time period in a virtual space ~200 μm under the retinal surface of the critical and fragile macula. Tremor data recorded during eye surgery have shown that it is present in the order of 100 um when transmitted to the tip of the instrument (12). Simulations in different settings come to the same conclusion (13). Thus, the level of ability required for such surgical procedure are literally at the human physiologic limit.

Furthermore, static positioning for controlled delivery of cells/gene vector solutions to the subretinal space as compared to dynamic motion present additional physiological challenges—holding static causes the appearance of micro jerks of 250 μm or more (13). All of these physiological constraints: tremor, jerks and low drifts are accentuated when attempting to remain stationary or when actuating an instrument (13).

Visual perception is the major source of information for the surgeon. This provides him with a three- dimensional representation of the surgical space, allowing him to estimate distances between instruments and target structures. While under optimal conditions, a 10 μm visual resolution can be achieved in XY (the planar field). In the Z axis, most important in depth perception, a particularly crucial element in subretinal delivery, observed resolutions are much lower (14).

Despite the emergence of intraoperative OCT (iOCT) technology which enhances a surgeon's ability to assess tissue depth, there are still practical limitations in iOCT systems including restricted OCT fields, shadowing of the operative site by intravitreal instruments and the inability for surgeons to operate and observe the iOCT image in real time. As previously shown, the iOCT is mostly beneficial during interruptions to assess the progress or completion of a surgical task (15–17).

### Reflux From the Subretinal Space Into the Vitreous Cavity

Subretinal injections using a transvitreal approach are widely used in clinical studies with viral vectors and cell suspensions through a 38–41G needle. Currently, a surgeon's success in accurately placing and estimating the volume of therapeutics delivered beneath the retina is based on surgical experience and en face visualization *via* the surgical microscope. The accepted practice for determining the volume of a subretinal bleb is by injection of a predetermined volume of the target therapeutic product from a calibrated syringe into the subretinal space. If the surgeon does not observe any leakage, it is assumed that all the injected volume is successfully delivered (18–21). However, based on volumetric estimation using the spherical cap formula of the detached area, 50% or less of the delivered volume reaches the target location (22). Direct volumetric measurements using intraoperative OCT showed that subretinal bleb size was on average 36% smaller than predicted by the surgeon using a dilute triamcinolone solution (23). In an experimental set-up for cell delivery to the subretinal space, 100% of cases had some degree of reflux (24).

**Figure 2:**
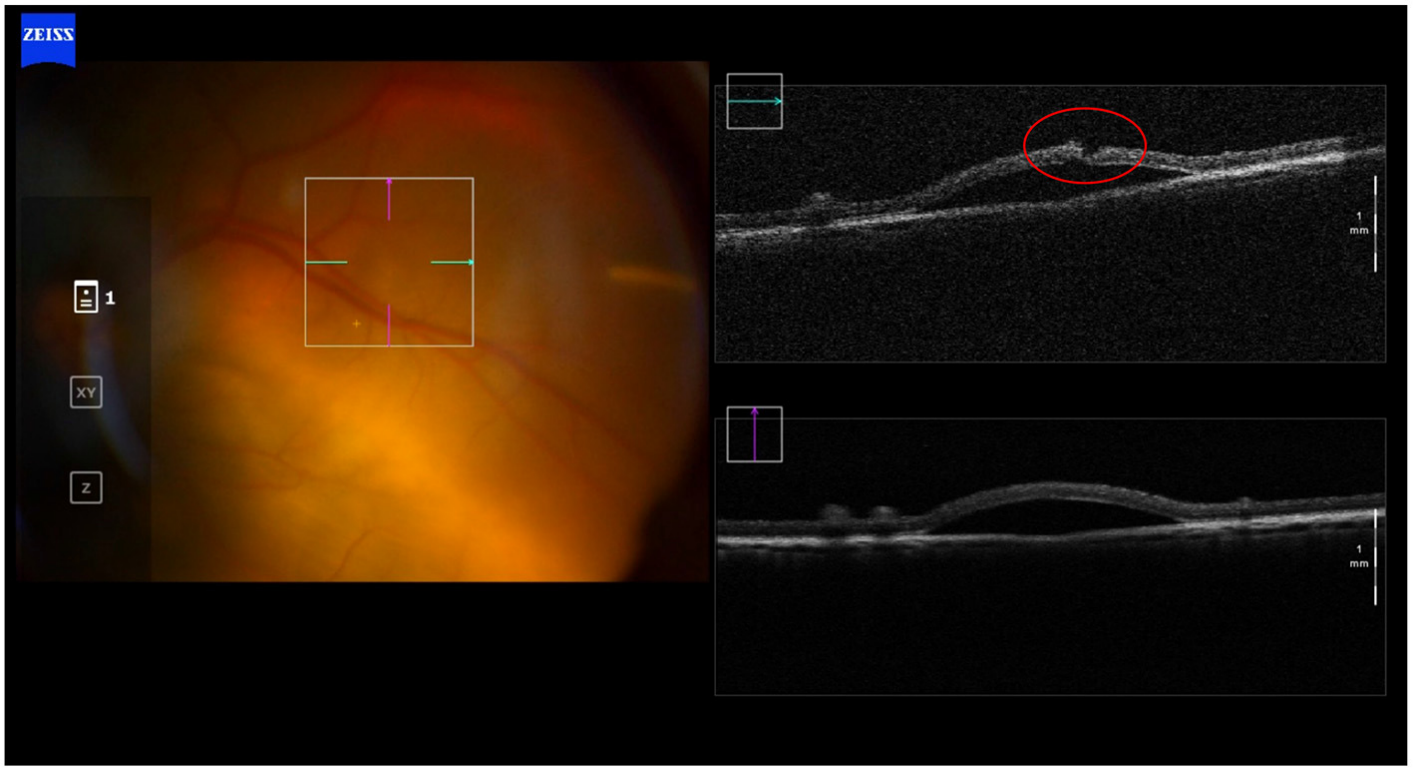
Transvitreal subretinal injection without pars plana vitrectomy. Intraoperative OCT picture showing absence of reflux after the subretinal injection in a non-vitrectomized living porcine eye. Intact vitreous may act as a plug and prevent reflux into the vitreous body, as we have observed in these subretinal injections experiments carried out in live pigs.

We have shown that removal of the needle from the subretinal space leads to leakage from the retinotomy as well as from the needle tip (25). The latter occurs as the built-up pressure in the catheter tubing (leading to the syringe) is released. Reflux from the retinotomy is reflective of tension within the bleb. Both phenomena are variable and can be minimized by prolonged retention of the needle tip in the subretinal space. Ideally, any reflux should be avoided as release of cells to the vitreous cavity can induce an immune response or lead to the formation of epiretinal membranes (20, 26–29). Intact vitreous can also act as a plug and prevent reflux into overlying retina, as we have observed in experiments carried out in live pigs. The necessity of performing a vitrectomy in all cases may require re-evaluation, even though this goes against the standard contemporary paradigms associated with vitreo-retinal surgery (Figure 2).

### Breaks in Bruch's Membrane and Loss of RPE Cells

Injection protocols dating to 2014 (30, 31) called for a 2-step procedure, which largely avoids causing a break in Bruch's membrane but does not completely eliminate the risk (20, 32). When breached, there is usually retraction of the underlying choroidal tissue leading to the formation of a small white round hole (20, 33). Two-step procedure increases the risk of having the retinotomy enlarge leading to more reflux, for this reason, a one-step approach has been favored in many clinical trials (34–36). The presence of an opening in Bruch's membrane increases the likelihood of transferring viral particles or exposing cells to the general circulation. Such an exposure can lead to a transient expression of gene products in blood or the formation of granulomas where cells are in contact with choroidal circulation (35, 36). In an experimental model of xenogeneic cells, transfected transchoroidally, granuloma formation was observed (though not with allogeneic cells) (33). A temporary, recordable immune response was noted in a patient reported by Schwarz et al. treated with cell therapy (20).

## Immune Consequence of Subretinal and Intravitreal Delivery

Altered, deviant immunity present in the subretinal space provides a certain degree of protection from inflammation and rejection but it does not prevent exposure to immunosurveillance mechanisms. Allogeneic fetal or pluripotent stem cells have both been known to cause an immunogenic response when injected intraocularly (37). The immune response varies depending on the mode of delivery (27). Indeed, clinical trials conducted using allogenic RPE cells for the treatment of AMD, 2 decades ago, were all met with graft rejection (38–42). Immune responses are also commonly observed using viral vectors and with mRNA transfections (42–44). The clinical features of these rejection episodes are often subtle and depend on the mode of delivery. They include inflammation in the anterior chamber and the vitreous, depigmentation at the site of injection, serous retinal detachment or retinal edema, mild to moderate cell infiltration around the cell transplant or in the overlying vitreous (seen on OCT) as well as mild vasculitis on fluorescein angiography (Table 2). Following an intravitreal injection, a dose dependent immune response is observed presenting as cells in either the anterior chamber and/or the vitreous. Both cellular and humoral factors have been implicated (45–47). In mice, an AAV injection into the vitreous leads to a transient mild spontaneously resolving inflammation but the total number of CD45+ T cells remains elevated, even weeks after the injection. Both innate and adaptive immunity play a role regardless of prior immune status (45). A patient treated for Lebers with a single intravitreal dose of 9 × 10^10^ vg and high pre-treatment titers of IgG neutralizing antibodies to AAV2, developed a significant post-injection ocular inflammation (46). Similarly, in April 2021, a patient enrolled in the INFINITY trial using Adverum's ADVM-022, an AAV.7m8 vector encoding a sequence for aflibercept developed hypotony, panuveitis and vision loss, 30 weeks following injection of 6 × 10^11^ vg (48). While improved pre-injection screening may reduce the incidence of such events, they limit the applicability of this approach. The presence of inflammation also will limit the efficiency of transfection or the duration of the effect if it leads to the elimination of transfected cells (45, 49, 50).

**Table 2:** Ocular manifestations associated with immune activation by RPE allografts in animal models and in human clinical trials.

**Diagnostic modality**	**Observations**
Visual acuity Visual field	Loss of visual function over the treated area
Intraocular pressure	Increased or hypotony (depends on severity of inflammatory response)
Color fundus imaging	Disruption of the transplant or targeted retinal structure Depigmentation Epiretinal membrane formation (ERM and macular pucker) Serous retinal detachment in the underlying RPE or around the transplanted area Encapsulation of the transplant Inflammatory vitreous opacity Inflammatory cell invasion in the treated area
Fluorescein angiography	Fluorescent leakage in the treatment area Retinal vasculitis
Indocyanine green angiography	Hypofluorescent dark spots in the choroid
Optical coherence tomography	Serous retinal detachment around the transplant or treated area Retinal edema around and over the area treat Sectoral nflammatory cell infiltration Vitreous cell infiltration Sectoral retinal disruption or of the transplant Disappearance of the outer retinal layers Epiretinal membrane Glial cell proliferation
Biomicroscopy	Anterior chamber cells and keratic precipitates

In contrast to the intravitreal route, transvitreal subretinal injections elicits less of an inflammatory reaction (51–53). Dose dependency has been observed both experimentally and clinically (54). In the Voretigene trials, 8% of patients showed signs of transient inflammation (52). At higher tiers and volumes, mild vitritis, optic disc swelling and some sheathing was observed several weeks after administration, and some focal pigmentary changes were observed 3.5 years later in the same patient (54). Hyper reflective foci were observed transiently in the retina of a patient treated with a low dose of AAV8, while at the intermediate dose, transient iridocyclitis was seen (55). Experimental studies confirm the lower immunogenicity of the transvitreal subretinal route but confirm that at higher doses (1 × 10^12^ vg for AAV8), inflammatory cell infiltration in the retina and choroid are observed in non-human primates (56). Subretinal injection do not seem to induce antibody production (57), also confirmed in another study by the group of R Ali, but only for low dose AAV injections. Indeed, higher doses lead to the expression of neutralizing antibodies that reduce the efficacy of repeated vector administration (58). At higher doses, the risk of a vitreous reflux increases, and a significant number of viral particles can persist despite flushing of the vitreous cavity with saline or BSS (59). Subretinal injections using transcleral micro needles lead to a diffuse peripheral expression when injected close to the pars plana but was associated with a localized inflammatory response consisting in the accumulation of macrophages and causing a localized chorioretinitis (60). When present, these responses appear 1–3 months after the therapy was applied (11). In the presence of inflammation, gene expression was noted to decrease progressively. Of note, even expression of AAV8 in the suprachoroidal space can be associated with a mild chorioretinitis and altered photoreceptor morphology (61).

Subretinal and intravitreal injections appear to lead to different phenotypes of immune response. With current vectors, the safest approach from an immune standpoint appears to be subretinal delivery, though this approach may not be appropriate if more superficial retinal cells such as ganglion cells are the targeted cell type. While the risk for an overall population may be small from an intravitreal approach, an immune response if induced can have devastating effects on vision as recently shown. These risks can be minimized by direct intraretinal targeting which can avoid priming the immune system and make use of the deviant immune response inherent to the retina and sub retinal space.

## Robotic Assistance for Subretinal Delivery

Recent advances in imaging and robotics can overcome the limitations listed above (7, 22). Robotic systems have been in routine use for more than 20 years in other surgical specialties. Advances in microrobotics make it now possible to perform highly delicate and precise surgeries such as the anastomosis of lymphatic vessels (62, 63). However, in gene / cell therapy, the inclusion of precision robotics to deliver accurately to a target location has only been explored to a limited extent. In obstetrics an article of 2016 has reported its successful use of a robot for the transplantation of frozen-banked ovarian tissue, and a proposal in 2021 suggests the use of a robotic platform to deliver stem cells into the brain of patients with Huntington's disease. The potential use of robots to treat other degenerative diseases is stimulating research into novel robotic platforms with for example MRI guidance of catheters to the spine, or even in cardiac surgery (64–67).

**Figure 3:**
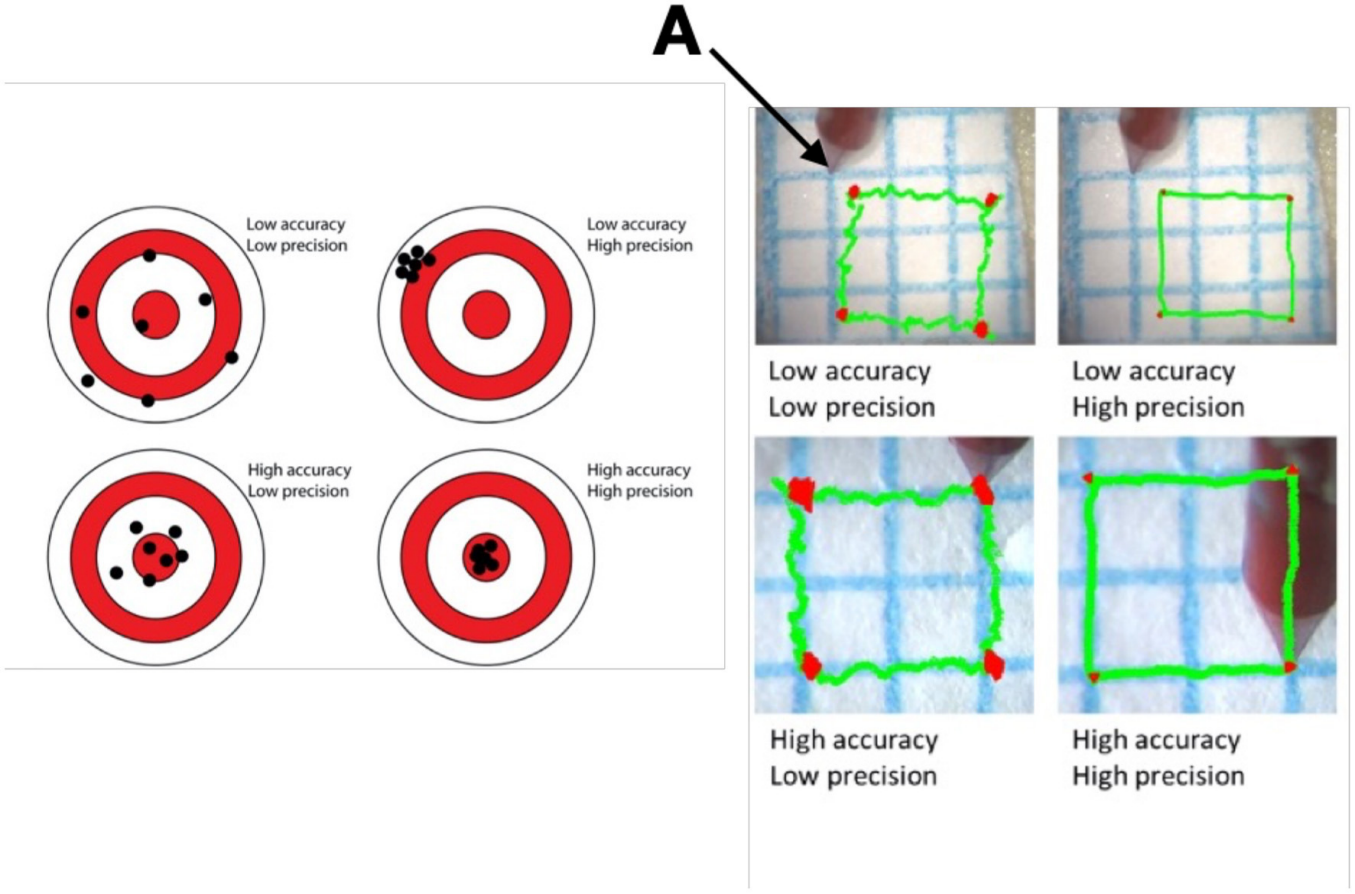
Difference in Precision and Accuracy in a schematic on the right and a dynamic task on the left. Picture and schematic representations of experiments using a laser vibrometer and video recording to assess the ability of a surgeon to superimpose or maintain the blue line with the tip of an instrument (A) held in hand. Precision refers to the degree of reproducibility of the motion, while accuracy refers to the contiguousness achieved in reference to an intended target.

In ophthalmology, high precision and accuracy are also required in the delivery of cell or gene products, and robotics can offer a solution. The terms precision and accuracy are often misunderstood. Precision refers to the degree of reproducibility of a motion, while accuracy refers to the contiguousness achieved in reference to an intended target This is best exemplified in Figure 3. Accuracy and precision can also be defined as components of both dynamic and static tasks of which the latter are more difficult to maintain. They can also be defined as a function of the axis (XYZ), and as shown in simulation experiments, the Z axis is the most demanding and where experience makes a difference (14). Unaided, dynamic precision in XY is about 40 μm for experienced and novice surgeons, and 35 vs. 60 μm in Z. Accuracy under the same conditions is between 68 and 87 μm for XY and 58–108 μm in Z. Robotics when self-actuated has an accuracy and precision between 1 and 3 μm.

Another issue is the location of the center of motion (RCM). Ideally it is placed at the site of insertion into the eye, but existing systems often have their RCM at a separate location (68). In Intuitive's da Vinci system, the RCM is located away from the eye making all intraocular movements less controllable and promotes unnecessary tension at the surface of the eye (69). Adapting existing robotic systems to ophthalmology is therefore fraught with problems (70).

Robotic systems specifically designed for intraocular surgery fall in three main categories: smart surgical tools, comanipulation and telemanipulation which are described more extensively in a review from 2018 (71). Table 3 summarizes advantages and drawbacks of different robotic designs for eye surgery in regard of subretinal delivery requirements. Master-slave systems allow a decoupling between the manipulation of an instrument from the surgeon's direct grip. Particularly in tele-manipulated systems, where the movement of the slave is controlled by a computer, this enables additional functions such as tremor filtering and an ability to introduce a variety of other commands that can lead to the precise positioning of the tip of a catheter at the appropriate depth under the retina. A catheter placed at the retinal surface, after an appropriate assessment of retinal thickness on an intraoperative OCT (iOCT), can be advanced to the exact required distance to place the tip of the instrument at the retinal RPE interface. It is the standby functionality the ability to suspend any task carried out by a robotic system that allows such fine measurements and provides the surgeon with the ability to carry out these precision task, uninhibited by time constraints. This independence means that the advancement of the needle through the retina can be planned once inside the eye in real time using existing iOCT machines, and because there is little motion of the eye, the signal to noise compensation algorithms are fully functional which allow for a fully optimized image of various retinal planes (72).

**Table 3:** Advantages and drawbacks of robotics designs for subretinal delivery.

	**Tremor filtering**	**Motion scaling**	**Eye stability**	**Surgical automation**	**Improved static tasks**	**Ergonomic control**	**Remote control option**	**Rapid exit**	**Predefined Boundaries to movements**	**Integration with iOCT**
Hand held	+	+/–	–	–	–	+	–	+	–	–
Comanipulator	+	–	+	–	+	–	–	–	+/–	–
Telemanipulator	+	+	+	+	+	+	+	+	+	+

**Figure 4:**
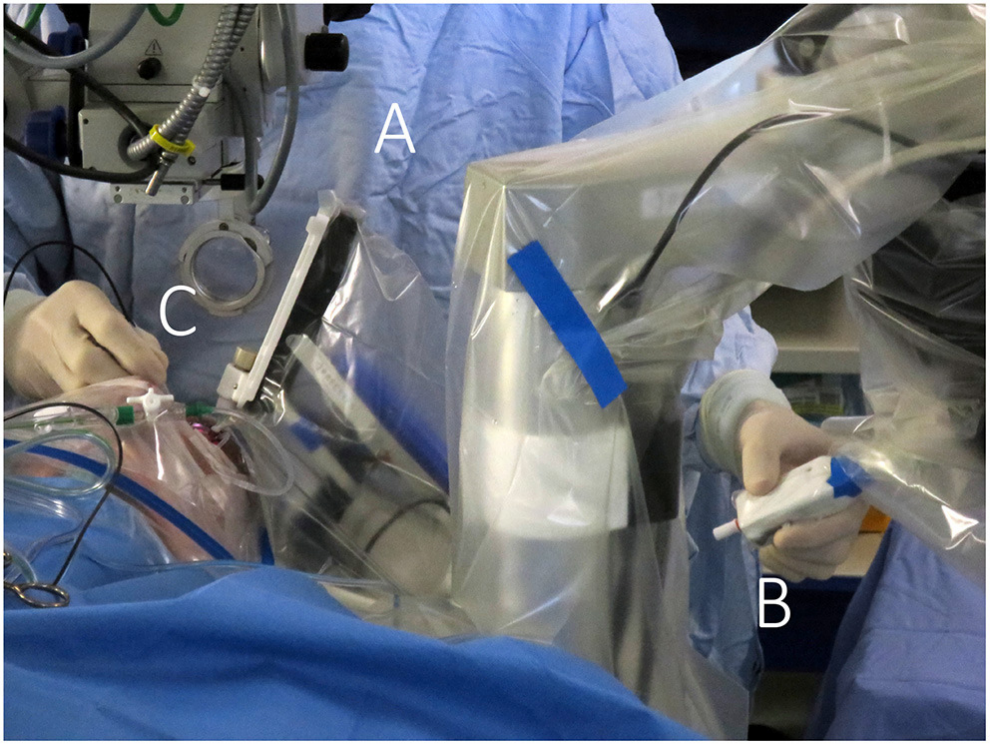
Telemanipulated robotic surgical system. The Preceyes surgical system (Preceyes bv, Eindhoven, The Netherlands) allows the surgeon to control a robotic instrument manipulator (A) located on the side of the headrest *via* a motion controller (B) held in one hand of the surgeon, while endoillumination is provided by a light pipe (C) held in the other hand. A particular advantage is the non-obstructive design of the robot, allowing for hybrid manual/robotic surgery and natural integration with the regular work flow of ophthalmic surgery sessions.

Robotics also gives researchers the ability to fully dissect a surgical maneuver to determine for subretinal injections for example the appropriate angle of penetration, depth, retinal contour and speed of injection that minimizes or prevents the risk of reflux. Such understanding and optimization were not possible prior to the use of robotics. By automating some of the steps, limiting the surgeon's interaction to steps which require his/her expertise such as choosing the appropriate location in the posterior pole and an adequate positioning at the retinal surface, the critical steps for the delivery of the cells or gene product to the subretinal space can be standardized (Figure 4).

**Figure 5:**
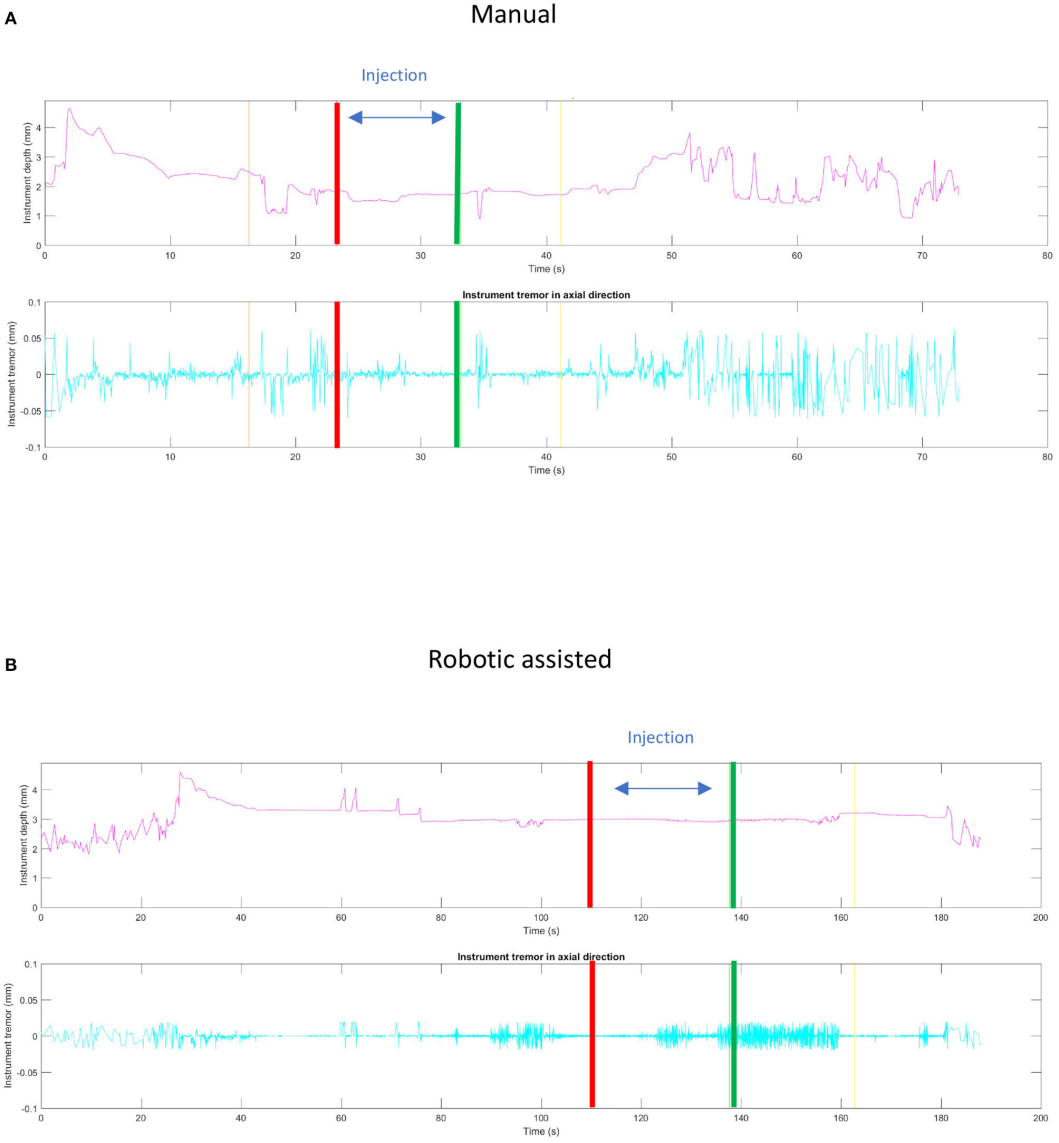
Comparison of static stability between manual **(A)** and robotic assisted **(B)** simulated subretinal injections. The red and green lines correspond, respectively, to the beginning and the end of the fluid injection. The ability to hold the instrument steadily at the point of insertion during this maneuver was measured to deviate around 40–266 μm, depending on the individual surgeon, when surgeries were performed manually as compared to a deviation of 1–2 μm with robotic assistance. Note that spikes in the robotic assisted procedure measurement were due to artifacts.

**Figure 6:**
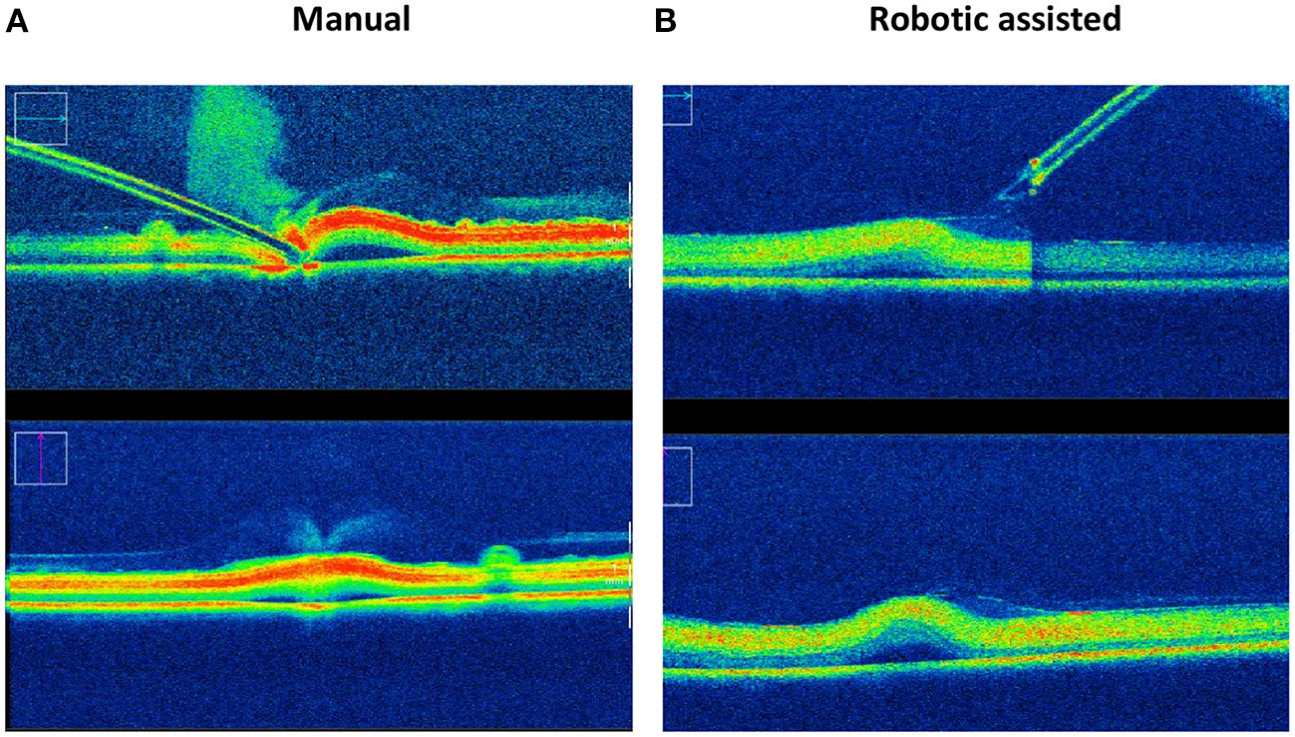
Reflux and bleb formation as seen by intraoperative OCT in an *ex-vivo* porcine model using a solution containing a contrast agent. Intraoperative OCT pictures of subretinal injection using an assistive robotic system **(B)** reduces the incidence of reflux from 100 to 20% and increases the rate of successful bleb formation from 40 to 100% in this porcine eye model, as compared to manual subretinal injection **(A)**.

This ability to reduce variability was clearly shown in an experiment carried out with retinal surgeons of various skill levels at a European retinal meeting (Euretina). Vitreoretinal surgeons, who had never used the PSS were asked to perform a simulated subretinal injection with and without the robotic device (7). A bleb was created more frequently with the use of the PSS (88 vs. 44%) with a reduction in the rate and severity of reflux (77 vs. 88%) was observed in this model that lacked any elastic tissue. Tremor was clearly reduced (Supplementary Video 1) when using the robot. The ability to hold the instrument steadily at the point of insertion ranged from 40 to 266 μm, depending on the individual surgeon, when surgeries were performed manually as compared to, 1–2 μm with robotic assistance (Figure 5) resulting in a diameter size reduction of the retinal hole. The use of the robotic arm also led to a longer “infusion time” ranging from 13 to 108 s, while the injections performed manually ranged from 18 to 85 s. In both cases, they had been instructed to inject over 20 s. These observations confirm studies performed in *ex-vivo* porcine eyes and *in vivo* data (73). In this same *ex-vivo* model, the use of PSS lead to the formation of subretinal blebs in 100% of cases, and reflux limited to 20% while manually, a bleb could only be created in 40% of cases with 100% reflux as seen on iOCT. Of note, to enhance visualization of reflux a contrast agent was added to the injection solution which allowed for the clear identification of reflux (Figure 6) (25).

## Next Steps to Robotic Assisted Surgery in the Subretinal Cell Therapy/Gene Therapy Environment

Microprecision robotic systems will require the key characteristics if they are to be used for ocular gene/cell therapy:

1. Precise positioning of a needle to the appropriate retinal/ subretinal layer.
2. Removal of any time constraints for delivery through positional stability.
3. Limitation of back flow and enlargement of the retinotomy by flow control and adaptive positioning.
4. Minimization of back flow on task completion.
5. A surgeon-friendly technique requiring minimal pre-operative training.
6. Task automation using appropriate software and intelligent tools.

While many of these points have been demonstrated in a non “gene therapy” setting (74), it is necessary to demonstrate the value in gene/cell therapy applications. Optimization of several functions will require further optimization with regards to retinal location and thickness. As each application and delivery location will require its own set of parameters. In essence for cell type, disease entity, intraocular location, specific proprietary software and hardware (intelligent devices) can be created.

The initial step will be to optimize parameters in live animals (pigs, rabbits, or monkeys) so that each injection achieves 90–95% of the objectives 1–5 listed above.

In a follow-up stage, incorporation of distant sensing and later pressure sensing would allow automation of the procedure. If coupled to an automatic infusion line, it could dynamically adapt to the degree of retinal stretch or relaxation as the bleb develops. In healthy retina, this is of course not needed, but ultimately, not only healthy retina of patients with genetic diseases will be targeted but also patients with thinned or scarred retinas, where it is difficult to appreciate the degree of tissue plasticity. At that stage, these further refinement in delivery may show that a full vitrectomy is superfluous.

## Conclusion

Safe, efficient and reproductible subretinal delivery of gene vector/cells solutions require skills which are literally at the limit of human dexterity.

Robotic assistance especially highly versatile telemanipulation robots can overcome these barriers. Such a tool would standardize the surgical procedure, increasing accuracy and precision resulting in a higher efficiency and safety and therefore better outcomes. It would also reduce the cost of clinical trials as the variability of drug delivery between surgeons as well as between centers will be significantly reduced. Costs of pre-clinical *in vivo* experiments can also be diminished for the same reasons. A standardized delivery system also facilitates the adoption of an approved drug as training of surgeons in the use of this delivery device can be limited in time and space and can be carried out with phantoms rather than live animals or ocular tissue.

## Author Contributions

All authors listed have made a substantial, direct, and intellectual contribution to the work and approved it for publication.

## Conflict of Interest

FW has been part of advisory boards for Abbvie, Allergan and Santen. MdS is Chief Medical Officer of Preceyes B.V., Eindhoven, Netherlands. He has received honoraria from Allergan, has been a part of advisory boards for Abbvie, Allergan, Janssen and Oxular Ltd. MdS was employed by MIOS SA. The remaining authors declare that the research was conducted in the absence of any commercial or financial relationships that could be construed as a potential conflict of interest.

## Publisher's Note

All claims expressed in this article are solely those of the authors and do not necessarily represent those of their affiliated organizations, or those of the publisher, the editors and the reviewers. Any product that may be evaluated in this article, or claim that may be made by its manufacturer, is not guaranteed or endorsed by the publisher.

## Supplementary Material

Supplementary Video 1 Manual vs. robot assisted simulated subretinal injection in an artificial retina model.
